# 3D bioartificial stretchable scaffolds mimicking the mechanical hallmarks of human cardiac fibrotic tissue

**DOI:** 10.36922/ijb.2247

**Published:** 2024-05-15

**Authors:** Mattia Spedicati, Francesca Tivano, Alice Zoso, Janira Bei, Mario Lavella, Irene Carmagnola, Valeria Chiono

**Affiliations:** 1 Department of Mechanical and Aerospace Engineering, Politecnico di Torino, Turin, Turin, Italy; 2 POLITO BioMedLab, Politecnico di Torino, Turin, Turin, Italy; 3 Interuniversity Centre for the Promotion of the 3Rs Principles in Teaching and Research (Centro 3R), Pisa, Italy; 4 Department of Management, Information and Production Engineering, University of Bergamo, Dalmine, Bergamo, Italy

**Keywords:** Bioartificial scaffold, Cardiac fibrosis, Stretchable scaffold, GelMA hydrogel

## Abstract

Human cardiac fibrotic tissues are characterized by a higher stiffness relative to healthy cardiac tissues. These tissues are unable to spontaneously contract and are subjected to passive mechanical stimulation during heart functionality. Moreover, scaffolds that can recapitulate the *in vivo* mechanical properties of the cardiac fibrotic tissues are lacking. Herein, this study aimed to design and fabricate mechanically stretchable bioartificial scaffolds with biomimetic composition and stiffness comparable to human cardiac fibrotic tissues. Poly(*ε*-caprolactone) (PCL) scaffolds with a stretchable mesh architecture were initially designed through structural and finite element method (FEM) analyses and subsequently fabricated by melt extrusion additive manufacturing (MEX). Scaffolds were surface functionalized by 3,4-dihydroxy-DL-phenylalanine (DOPA) polymerization (polyDOPA) to improve their interaction with natural polymers. Scaffold pores were then filled with different concentrations (5%, 7%, and 10% w/v) of gelatin methacryloyl (GelMA) hydrogels to support *in vitro* human cardiac fibroblasts (HCFs) 3D culture, thereby producing bioartificial PCL/GelMA scaffolds. Uniaxial tensile mechanical tests in static and dynamic conditions (1 Hz; 22% maximum strain) demonstrated that the bioartificial scaffolds had *in vivo*-like stretchability and similar stiffness to that of pathological cardiac tissue (tailored as a function of the number of PCL scaffold layers and GelMA hydrogel concentration). *In vitro* cell tests on bioartificial scaffolds using HCF-embedded GelMA hydrogels under static conditions displayed increasing cell viability, spreading, and cytoskeleton organization with decreasing GelMA hydrogel concentration. Moreover, *α*-smooth muscle actin (*α*-SMA)-positive cells were detected after 7 days of culture in static conditions followed by another 7 days of culture in dynamic conditions and not in HCF-loaded scaffolds cultured in static conditions for 14 days. These results suggested that *in vitro* culture under cyclic mechanical stimulations could induce an HCF phenotypic switch into myofibroblasts.

## 1 Introduction

Cardiovascular diseases (CVDs) are the leading cause of morbidity and mortality worldwide.^1^ Among them, myocardial infarction (MI) causes the irreversible loss of billions of cardiomyocytes, followed by cardiac fibrosis and progressive heart failure.^1^ From weeks to months post-MI, cardiac fibrotic scar forms in the infarcted area, consisting of a stiff extracellular matrix (ECM), populated with cardiac fibroblasts.^2^ Predictive *in vitro* models of the human pathological cardiac tissue are highly demanded to support the *in vitro* preclinical validation of new therapeutic approaches, thereby minimizing *in vivo* trials on animal models and aligning with the 3Rs principle (i.e., replacement, reduction, and refinement).

Due to the high socioeconomic impact of ischemic heart disease, biomimetic scaffolds are highly demanded for the future *in vitro* engineering of human cardiac fibrotic tissue. An infarcted human heart is characterized by the presence of a stiff ECM that is rich in type I and III collagens.^2^ Natural polymer hydrogels, including gelatin methacryloyl (GelMA), have been frequently used to support the *in vitro* culture of human cardiac fibroblasts (HCFs).^5–8^ Sadeghi *et al*. employed GelMA hydrogels cultured with cardiac fibroblasts from neonatal rats to study pathological remodeling occurring during cardiac fibrosis. Different GelMA hydrogel concentrations (5%, 7%, and 10% w/v) and degrees of methacryloyl substitution (medium [53%] and high [81%]) were used in the study.^3,4^ However, hydrogels lack structural biomimetic cues for cell guidance, cannot bear cardiac tissue-like cyclic mechanical deformations, and have short degradation times.^5^ Synthetic polymer scaffolds could overcome the main limitations of natural polymer hydrogels, as they have superior stability in physiological media, may withstand cardiac tissue-like cyclic deformation, and provide topographical cues to cells. Among the synthetic polymers, thermoplastic polyesters, such as poly(*ε*-caprolactone) (PCL), have been widely used in tissue engineering. PCL is a biocompatible and bioresorbable polymer approved by the United States (US) Food and Drug Administration (FDA).^3^ PCL is an optimal polymer for scaffold manufacturing by melt-processing techniques, owing to its low melting temperature (approximately 60 °C), which minimizes thermomechanical degradation during scaffold fabrication. As PCL is not elastomeric, PCL scaffold architecture can be designed to obtain stretchable substrates.^5–8^ Indeed, Olvera *et al*.^6^ fabricated PCL-based cardiac patches with high stretchability through a missing-rib unit network. Castilho *et al*.^12^ fabricated PCL-based scaffolds for cardiac tissue engineering with rectangular-shaped mesh geometry, later improved through a hexagonal-shaped mesh geometry to best reproduce the native honeycomb microstructure of the myocardium.^6,7^ Paxton *et al*.^9^ employed bow-tie architecture for PCL stretchable tubular scaffolds, while Bas *et al*.^10^ and Saidy *et al*.^11^ investigated the mechanical tunability of PCL scaffolds provided with filaments with a wavy arrangement. PCL scaffolds have been previously used for the development of *in vitro* healthy cardiac tissue models^11^ or as implantable cardiac patches.^6^ Such platforms were fabricated by melt electrospinning writing (MEW), an additive-manufacturing (AM) technique that enables the production of polymeric scaffolds with complex geometries, based on filaments of a few microns in size. However, MEW scaffolds have an increased frequency of defects with increasing thickness due to charge interference between the nozzle and deposited layers.^12,13^ Melt-extrusion additive manufacturing (MEX) is a simpler and more rapid prototyping approach that fabricates reproducible scaffolds by controlled deposition of molten polymer filaments.^14,15^ Polymer filaments in MEX scaffolds generally have a larger size than the polymer filaments in MEW scaffolds (≥100–150 µm vs. ≤50–20µm,^3,14^ respectively). To date, MEX has been mainly applied to prepare stiff PCL scaffolds with straight filaments and square-meshed geometry for subsequent *in vitro* cell cultures.^16^ As human cardiac fibrotic tissues are incapable of spontaneous cyclic contraction, they are typically subjected to passive mechanical stimulation during heart-beating activity.

In this study, we fabricated and characterized 3D stretchable PCL scaffolds with wavy aligned filaments by MEX after an accurate structural and mechanical scaffold design by an analytical method and the finite element method (FEM).^18^ Despite their superior processability and mechanical properties over natural polymers, synthetic polymers lack cell adhesive cues. In contrast, 3D hydrogels based on natural polymers support cell attachment but generally exhibit a limited ability to withstand cyclic mechanical stretching.^19–21^ “Bioartificial” substrates, combining synthetic and natural polymers, may thus represent an optimal option, as they synergistically combine the promising properties of both polymer types.^17–21,25^ Embedding the natural polymer hydrogel into a stretchable synthetic polymer scaffold improves its mechanical resistance and structural stability during cyclic mechanical stimulation of the whole construct.^19^ Therefore, bioartificial scaffolds were prepared by filling PCL scaffold pores with 5%, 7%, and 10% w/v GelMA hydrogels, selected based on previous literature.^3^ The elastic modulus of the bioartificial scaffolds could be modulated by hydrogel filler mechanical properties to mimic the mechanical properties of human cardiac fibrotic tissues. In detail, the reference values for Young’s modulus of human cardiac fibrotic tissues (measured by tensile tests) range from 400 kPa to 9 MPa as a function of patient sex, age, and pathophysiological conditions of the left ventricle.^22,23^ In contrast, diastolic elastic deformation ranges at 10–22%.^21–23^ The designed mechanically stretchable bioartificial scaffolds supported the *in vitro* 3D culture of HCFs. Dynamic *in vitro* cell culture studies using MechanoCulture T6 bioreactor displayed markers of cardiac fibrosis that could suggest HCFs activation into myofibroblasts. This work highlighted the potential of stretchable bioartificial scaffolds for human cardiac fibrotic tissue engineering by *in vitro* culture of HCFs under cyclic mechanical stimulation. In comparison to miniaturized cardiac fibrotic tissue models on a chip,^29^ which are limited to the *in vitro* preclinical testing of drugs and nanotherapeutics, the present cardiac scar model could also be exploited for the testing of medical devices and advanced therapies based on scaffolds/hydrogels under dynamic mechanical stimulation. Future applications by our group will include the *in vitro* preclinical study of cardiac regenerative therapies under dynamic conditions, such as the direct reprogramming of HCFs into cardiomyocytes.^24,30,31^

## 2 Methods

### 2.1 Materials

PCL pellets with a molecular weight of 43,000 Da were purchased from Polysciences GmbH (Germany). GelMA (60% degree of methacryloyl substitution and a gel strength of 300 g Bloom), lithium phenyl-2,4,6-trimethylbenzoylphosphinate (LAP), and 3,4-dihydroxy-DL-phenylalanine (DOPA) were purchased from Sigma-Aldrich (USA). Phosphate buffered saline (PBS) was purchased from Thermo Fisher Scientific (USA), while HCFs and fibroblast growth medium-3 (FGM-3) were purchased from PromoCell GmbH (Germany).

### 2.2 Poly(*ε*-caprolactone) mesh geometry

**Figure 1:**
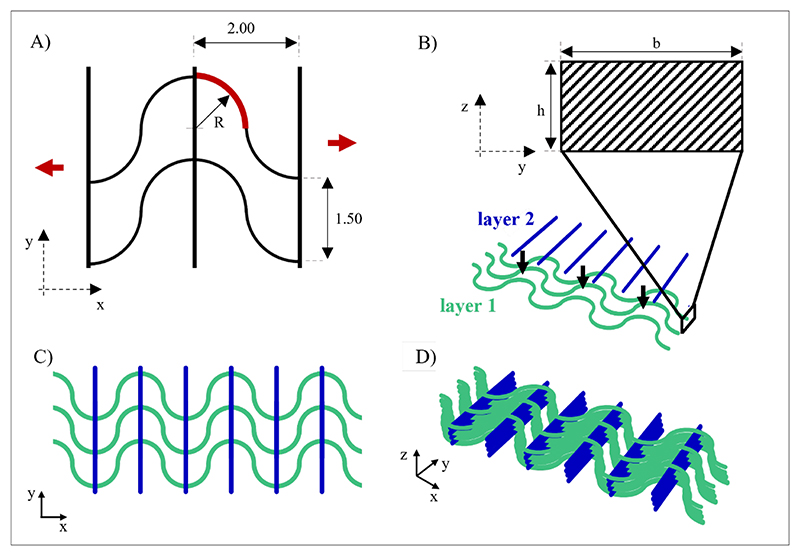
Poly(*ε*-caprolactone) (PCL) mesh geometry. (A) The geometry of a stretchable PCL mesh unit cell model. Red arrows in the *x*-direction indicate the direction of mechanical stretching applied to the mesh; the element portion considered for structural analysis is highlighted in red; spacing dimensions, expressed in mm, and radius (*R*) are reported. (B) Single filament cross-section and schematic representation of the superimposition of layers 1 and 2. (C) The 2D mesh geometry with layers 1 and 2. (D) The 3D mesh geometry is obtained by the superimposition of the eight layers (from layers 1 to 8), corresponding to four of the 2D mesh structures in (C).

A computer-aided design (CAD) model of the scaffold was designed based on eight superimposed layers (0.15 mm thickness per layer) (Figure 1). The geometry was based on the alternation of two superimposed layers: the first layer made of wavy equidistant filaments aligned along the *x*-direction and the other layer consisting of straight parallel filaments aligned along the *y*-direction (Figure 1). The wavy pattern was composed of a sequence of half semicircles with a radius (*R*) of 1 mm (Figure 1A). The inter-fiber distance between two close wavy and straight filaments in each layer was 1.5 and 2 mm, respectively (Figure 1A). This was considered as the unit cell for the stretchable PCL mesh. The cross-section of each filament was approximated to a rectangle with an area *b* × h derived from scanning electron microscopy (SEM) and optical microscopy analyses (Figure 1B). The 2D mesh model with two layers and the 3D mesh model with eight layers are displayed in Figure 1C and D, respectively.

### 2.3 Mechanical modeling

#### 2.3.1 Structural analysis of scaffold stiffness

**Figure 2:**
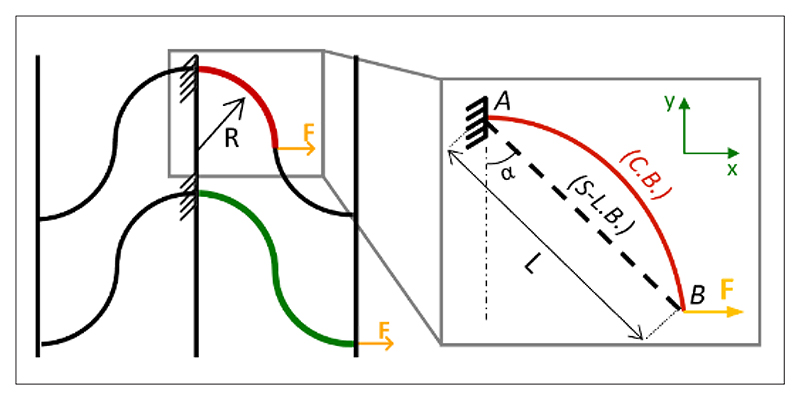
Straight-line beam (S-L.B.) and curved beam (C.B.) approximations. *F* is the applied force and *M* is the bending moment.

Structural analysis aimed to define the proper PCL mesh design in terms of thickness (number of layers) to ensure cardiac tissue-like stiffness and stretchability under physiological mechanical stimulation (i.e., Young’s modulus between 400 kPa and 9 MPa, and bearing an elastic deformation ≤22%).^21–23^ PCL mesh stiffness was calculated considering the repeating half-semicircle element of the wavy pattern (Figure 1A) approximated with a straight-line beam (S-L.B.) or a curved beam (C.B.) (Figure 2). The stiffness of a single element in the mesh was calculated:

(1) $$K_{ij}=F/\delta$$

where *F* denotes the applied force and *δ* is the displacement, both of which are computed considering S-L.B. and C.B. approximations, respectively (Equations SIII and SIV). Calculated stiffnesses were considered a property along the *x*-axis according to the force (*F*) direction (Figure 2). In the case of S-L.B. approximation (Table 1), stiffness was calculated using displacement computed through the elastic line equation. On the other hand, in the case of the C.B. approximation (Table 1), stiffness was computed through Castigliano’s theorem,^23^ by determining the displacements due to tensile and bending contributions.

**Figure 3:**
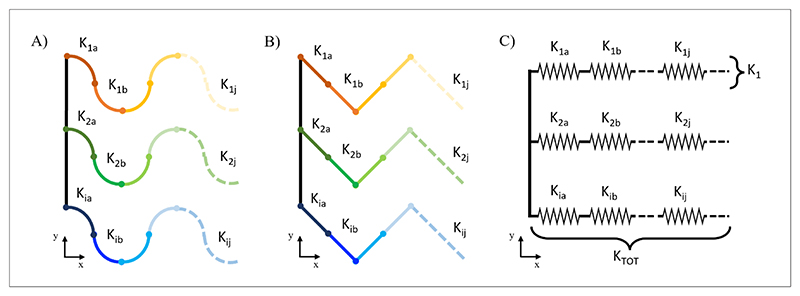
Single elements for total stiffness evaluation considering (A) C.B. and (B) S-L.B. approximations, respectively. (C) 2D mesh model approximation to a spring with a defined stiffness. Abbreviations: C.B.: Curved beam; S-L.B.: Straight-line beam.

By considering a single beam element as a spring, each wavy filament is a spring series along the horizontal direction while their assembly along the vertical direction is a spring parallel (Figure 3A–C). Total scaffold stiffness was estimated by calculating the stiffness of a single wavy filament made of *j-beam* elements in series and multiplying this value by the *i* value of filaments along the vertical direction (Table 2).

#### 2.3.2 Finite element method analysis of poly(ε-caprolactone) scaffolds

The mechanical behavior of the PCL scaffolds was investigated using 1D static structural FEM via the Ansys Workbench R22.1 software. In detail, FEM simulation was used for computing displacement behavior under tensile force and consequent stiffness evaluation. Moreover, Von Mises stress distribution was displaced to evaluate stress accumulation points. Since the construct was intended to be used within its elastic field limit, analysis was conducted assuming linear elastic material properties. Moreover, the model of the material was assumed to be linear and isotropic due to material homogeneity through the filament cross-section, while load distribution was distributed with stress applied along the wavy filaments’ direction. For each analyzed stacking configuration with a different number of layers (alternating wavy and straight filaments in Figure 1B), a CAD model was prepared, consisting of beams discretized into 1D FEM mesh made of “BEAM188” elements (from the Ansys library). Specimen dimensions were set to 14 × 4.5 mm^2^, resulting in a total of 3.5 unit cells along the *x*-axis and 1.5 unit cells along the *y*-axis (considering the dimensions in Figure 1A and configuration in Figure 1C). Poisson’s ratio was derived from previous literature (*v* = 0.3).^32^

**Figure 4:**
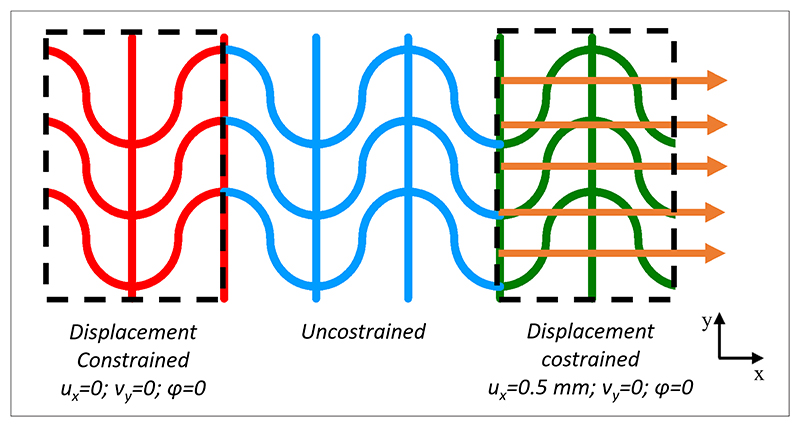
Boundary conditions of the FEM model were applied on stretchable PCL specimens for experimental analysis. Abbreviations: FEM: Finite element method; PCL: Poly(*ε*-caprolactone).

A fixed support boundary condition of constrained displacement was imposed on a scaffold length corresponding to one unit cell length along the *x*-axis on one scaffold end, limiting both translational and rotational degrees of freedom (Figure 4). An external load, in the form of an imposed displacement, was applied to one unit cell length on the free side of the scaffold, simulating the action of a tensile force to stretch the scaffold along the *x*-direction. A central unconstrained scaffold portion with a total length of 1.5 unit cells was allowed to undergo deformation, representing the effective tested specimen. The imposed displacement along the *x*-direction (same as the F application) was 0.5 mm under the hypothesis of linear elastic deformation. Stiffness was calculated as the ratio between force and displacement (Equation 1).

To optimize the accuracy of FEM results and computational requirements for the simulations, a FEM mesh convergence study was performed by varying the refinement of the mesh during solution estimation. As the scaffolds were subjected to previously described boundary conditions, tensile force acting on a single beam was computed using an increasing number of nodes.

### 2.4 Fabrication of poly(*ε*-caprolactone) scaffolds

Based on the aforementioned mesh design, PCL scaffolds were produced through MEX using a commercially available 3D printer (ROKIT Invivo 3D Printer Premium, ROKIT Healthcare, Korea). Different sizes of PCL scaffolds were fabricated for structural and FEM analyses and mechanical tests: 6 × 4.5 mm^2^ with thickness of 0.3 mm for cell tests; 14 × 4.5 mm^2^ with thickness varying according to the number of layers (from two to eight layers). PCL pellets were loaded in a heated print head and melted at 100 °C. By applying compressed air with a pressure of 550 kPa, filaments were extruded through a nozzle with a diameter of 200 µm. The distance between the nozzle and the printing bed was customized through a G-code to achieve suitable print quality and resolution.

### 2.5 Preparation of photocurable gelatin methacryloyl hydrogels

GelMA prepolymer solution was prepared at three different concentrations (5, 7, and 10% w/v) in FGM-3 and stirred at 50 °C in dark conditions for 1 h. LAP was added (0.5% w/v) to the GelMA prepolymer solution and stirred for 15 min at 50 °C. Different volumes of GelMA solutions (30–300 µL) were prepared accordingly, and GelMA hydrogels were then formed by ultraviolet (UV) irradiation (wavelength: 365 nm; power density: 10 mW/cm^2^) using a flexible light guide (Hamamatsu LC8 lamp, Hamamatsu Corporation, Japan). Light intensity was measured with a UV radiometer (UV Power Puck II, EIT, USA). GelMA hydrogels at the three different concentrations were coded as GelMA_5, GelMA_7, and GelMA_10, respectively.

### 2.6 Preparation of bioartificial scaffolds

PCL scaffolds were surface functionalized by a mussel-inspired approach, using DOPA to improve interfacial adhesion with GelMA hydrogels as previously reported.^33^ GelMA solutions, with a volume ranging from 30 to 200 µL depending on the PCL scaffold size (i.e., 6 × 4.5 mm^2^ with a thickness of 0.3 mm or 14 × 4.5 mm^2^ with thickness varying according to the number of layers) were then poured into the pores of polyDOPA-functionalized PCL scaffolds.

The scaffolds were then cured by UV irradiation (365 nm) at specific times based on the hydrogels’ rheological properties (30 s for GelMA_5 and GelMA_7; 45 s for GelMA_10) (see section 2.9.). The bioartificial scaffolds were coded as PCL/GelMA_5, PCL/GelMA_7, and PCL/GelMA_10, depending on the hydrogel concentration.

### 2.7 Mechanical characterization

#### 2.7.1 Uniaxial tensile tests

The mechanical behavior of PCL and PCL/GelMA scaffolds was evaluated through uniaxial tensile tests in both dry and wet conditions using MTS QTest/10 MTS® System Corporation, Eden Prairie, MN, USA. Initially, tensile tests were carried out on straight PCL filaments fabricated by MEX (0.15 mm diameter and 25 mm length), using a load cell of 10 N and a velocity of 5 mm/min. These tests were performed to evaluate the Young’s modulus (*E*) of a single PCL filament and subsequently used for analytic and FEM analyses. Tensile tests were also performed on dry PCL scaffolds with 3.5 × 1.5 unit cells in the *x*–*y* plane (corresponding to 14 × 4.5 mm^2^), a gauge length of 1.5 unit cells along the *x*-axis (corresponding to 6 mm), and a thickness depending on the number of layers (0.3–0.9 mm). A load cell of 10 N and a velocity of 1 mm/min were used. Force–elongation and stress–strain curves were plotted to calculate the stiffness and Young’s modulus of samples, respectively.

Based on the results of the PCL mesh tensile tests, bioartificial PCL/GelMA scaffolds (3.5 × 1.5 unit cells) with four and eight layers were characterized by uniaxial tensile tests in wet conditions using the same set-up previously described. Similar to that of PCL scaffolds, force–elongation and stress–strain curves were plotted and analyzed to calculate stiffness and Young’s modulus of samples, respectively, in wet conditions.

In detail, stiffness (a parameter depending on scaffold mesh thickness and geometry) was measured by the 0.2% offset method from force–elongation curves by constructing a parallel line to the linear segment. Likewise, Young’s modulus of the PCL/GelMA scaffold was determined from the stress–strain curve slope by applying the 0.2% offset method at the initial linear region and optimizing the PCL/GelMA scaffold cross-section as the bulk thickness. All samples were tested in triplicates.

#### 2.7.2 Cyclic uniaxial tensile tests

The PCL/GelMA scaffolds were subjected to cyclic uniaxial tensile tests in wet conditions using MTS QTest/10 under 4% pre-deformation. The samples were then subjected to cyclic mechanical deformations with sinusoidal waveform up to 22% maximum deformation and at 1 Hz frequency for overall 100 cycles. Force–elongation and stress–strain curves were plotted to validate the elastic deformation behavior of PCL/GelMA scaffolds.

### 2.8 Morphological evaluations

PCL scaffold and freeze-dried GelMA hydrogel morphologies were observed by optical microscopy (Leica Z16 APO A Microscope, Leica Microsystems, Germany) and SEM (Tescan Vega Microscope, Tescan, USA) at different magnifications. Before SEM analysis, the samples were coated with a thin gold layer (Q150S, Quorum Technologies, United Kingdom [UK]). The ImageJ software was employed for image analysis of PCL scaffolds (top view and section) and freeze-dried GelMA hydrogels (section).

### 2.9 Physicochemical characterization of photocurable gelatin methacryloyl hydrogels

Photo-rheology was performed to investigate the kinetics and time of photocrosslinking the three different concentrations of GelMA hydrogels in response to UV and visible (Vis) light irradiation. A modular compact rheometer (MCR 302, Anton Paar, Austria) was equipped with parallel plates and a UV (bulb type: 365 nm; power density: 18 mW/cm^2^; Hamamatsu LC8 lamp) or Vis (bulb type: 400–700 nm; power density: 18 mW/cm^2^; Hamamatsu LC8 lamp) light source under the bottom quartz plate.^34^ The gap between the two plates was set at 0.8 mm. The UV or Vis lamp was switched on 60 s after the start of the test to allow system stabilization before the beginning of the crosslinking process. After that, GelMA hydrogel solutions were exposed to irradiation for 3 min to evaluate their curing time. All the measurements were performed at a constant temperature (37 °C), with steady rotational oscillation at a frequency of 1 Hz and a constant strain amplitude of 1%.

The stability tests were evaluated in terms of dissolution behavior. GelMA_5, GelMA_7, and GelMA_10 hydrogels were dissolved in 200 μL water and subsequently in 400 μL sterile PBS) at 37 °C at different time steps (3, 7, and 14 days). PBS was refreshed every 3 days, keeping the solution sterile. At the end of each time point, the samples were washed with MilliQ water and then frozen at −20 °C. GelMA_5, GelMA_7, and GelMA_10 samples (in triplicate) were then freeze-dried and weighed. The dissolution degree (DD) was defined as:

(2) $$DD(\text{\%})=\left(\left(w_{0}-w_{f}\right)/w_{0}\right)\times 100$$

where *w*_*f*_ is the weight of incubated hydrogels after freeze-drying at each time point with respect to the initial weight (*w*_0_).

### 2.10 Gelatin methacryloyl hydrogel cytocompatibility

Following the ISO 10993 regulation, a cytocompatibility assay was performed on eluates from GelMA_5, GelMA_7, and GelMA_10 hydrogels.^34^

HCFs from the human ventricle were extracted and cultured with FGM-3 in a humidified incubator at 37 °C and 5% CO_2_. After trypsinization with 0.25% trypsin-ethylenediaminetetraacetic acid (EDTA) (Life Technologies, USA), HCFs were seeded in a 96-well plate with a cell density of 10^4^ cells/well for 24 h to reach confluence and cultured in FGM-3 medium. Then, the culture medium was replaced with a conditioned medium, previously obtained by submerging 100 mg of GelMA_5, GelMA_7, and GelMA_10 hydrogels (prepared in FGM-3) in 1 mL of FGM-3 at 37 °C for 24 h. Before *in vitro* cell tests, the conditioned medium was sterilized by filtration through a sterile Biofil syringe filter with a 0.22 µm pore size. In the control samples, the culture medium was also FGM-3. After 24 h of cell culture, the medium was replaced with 100 µL of non-fluorescent resazurin solution composed of CellTiter-Blue Cell Viability Assay reagent (Promega, USA), diluted to 1:10 in the new FGM-3. Cell viability was evaluated after a 4 h incubation in terms of fluorescence intensity measured by a microplate reader (BioTek Instruments, USA) at excitation (ex) and emission (em) wavelengths of 560 and 590 nm, respectively. Six measurements were performed for each hydrogel concentration to calculate the mean values and standard deviations compared to control samples.

### 2.11 Long-term cell viability and cytotoxicity tests on bioartificial stretchable scaffolds

PolyDOPA-PCL scaffolds with two layers (6 × 4.5 mm^2^, considering the gauge length used in tensile tests) were sterilized by incubation in 70% ethanol solution for 30 min and subsequently dried in a biological hood before cell tests.

Sterile GelMA_5, GelMA_7, and GelMA_10 solutions were prepared as follows: (i) GelMA and LAP powders were UV-sterilized for 30 min in a biological hood (MSC-Advantage Class II Biological Safety Cabinet, Thermo Fisher Scientific); (ii) GelMA powders were mixed with sterile FGM-3 and incubated at 50 °C under stirring for 2 h until complete GelMA dissolution; (iii) LAP powder was added to each GelMA solution in the sterile hood and the system was stirred at 50 °C for 15 min in dark conditions.

HCFs were detached from the Petri dishes using trypsin-EDTA and centrifuged to obtain cell pellets with defined amounts of cells (1,000,000 cells/mL), which were then added to the GelMA_5, GelMA_7, and GelMA_10 solutions containing LAP at 37 °C. Cells were encapsulated by mixing GelMA solutions with cell pellets. Cellularized GelMA solutions (30 µL) were deposed into polyDOPA-PCL scaffold pores and on 48-well plates as the control and cured under UV irradiation at specific times based on the hydrogels’ rheological properties (30 s for GelMA_5 and GelMA_7; 45 s for GelMA_10). After curing, viability and cytotoxicity tests were performed.

Live/Dead Cell Viability assay (Life Technologies, USA) was performed after 1, 7, and 14 days. Briefly, cellularized GelMA hydrogels cultured in 48-well plates were treated for 30 min with Live/Dead reagent (Life Technologies, USA), composed of 2 µM calcein acetoxymethyl (AM) and 4 µM ethidium homodimer-1 diluted in PBS. Samples were then imaged with a Nikon Ti2-E fluorescence microscope (Nikon Instruments, Japan).

Moreover, CellTiter-Blue Cell Viability Assay and CytoTox-ONE Homogeneous Membrane Integrity Assay (Promega, USA) were performed after 7 and 14 days of culture. CellTiter-Blue solution (300 µL) was incubated for 6 h with cellularized GelMA and PCL/GelMA samples, while CytoTox-ONE assay was performed on culture medium from both samples following manufacturer instructions (see section 2.10.).

PCL/GelMA samples (with the three different GelMA concentrations) were cellularized with HCFs and cultured for 14 days in static conditions to assess the effect of GelMA concentration on cell viability, spreading, and cytoskeleton morphology. After selecting the optimum GelMA concentration for cell spreading and cytoskeleton conformation, *in vitro* cell tests were repeated under mechanical stimulation to assess its influence on HCF activation into myofibroblasts. PCL/GelMA samples were cultured for 7 days in static conditions (based on resultant cell morphologies within GelMA hydrogels in static conditions) and mechanical stimulation was subsequently applied to the scaffolds for 7 additional days. The MechanoCulture T6 bioreactor (CellScale, Canada) was employed to cyclically stretch the PCL/GelMA samples (4.5 × 1.5 unit cells) in the *x*–*y* plane (corresponding to 18 × 4.5 mm^2^) with a gauge length of 1.5 unit cells along the *x*-axis (corresponding to 6 mm) and a four-layer thickness. The samples were subjected to cyclic mechanical deformations up to 10% maximum deformation (1 mm) at 1 Hz frequency and incubated in FGM-3 at 37 °C. The experimental setup is displayed in Figure S6 and Video S1, Supporting Information.

### 2.12 Immunofluorescence

Cellularized PCL/GelMA scaffolds were fixed in 4% w/v paraformaldehyde (Alfa Aesar, USA) in PBS for 15 min and subsequently washed with PBS. The cells were permeabilized with Triton X-100 (Sigma-Aldrich, USA) 0.5% in PBS for 10 min. The samples were then blocked with 2% bovine serum albumin (BSA, Sigma-Aldrich, USA) in PBS for 30 min, followed by either (i) staining with rhodamine-phalloidin (Life Technologies, USA) or (ii) incubation with primary antibodies (i.e., anti-actin smooth muscle [*α*-SMA; A7607, Sigma-Aldrich, US], anti-fibronectin [F3648, Sigma-Aldrich, USA], anti-collagen I [2456, Sigma-Aldrich, USA], and anti-collagen III [SAB4500367, Sigma-Aldrich, USA]), and secondary antibodies (i.e., anti-mouse Alexa Fluor 555 [Life Technologies, USA] or Alexa Fluor 488 [Life Technologies, USA]) diluted in 2% w/v BSA in PBS. Nuclei were counterstained with 4′,6-diamidino-2-phenylindole (DAPI; Sigma-Aldrich, USA). Samples were imaged using the Nikon Ti2-E fluorescence microscope (Nikon Instruments, Japan). Immunofluorescence experiments were performed in biological triplicates.

### 2.13 Statistical analysis

Statistical analysis of variance (ANOVA) was performed using GraphPad Prism version 9.0.0 (GraphPad Software Inc., USA).

## 3 Results and discussion

In this work, bioartificial PCL/GelMA scaffolds were designed and fabricated. These scaffolds were able to reproduce the stiffness of human cardiac fibrotic tissue to sustain *in vivo*-like cyclic mechanical deformations and support HCF culture. The ability of bioartificial scaffolds to sustain cyclic mechanical deformations was imparted by the PCL scaffold architecture, while stiffness could be adjusted by both the number of PCL scaffold layers and the concentration of GelMA hydrogels. Notably, the GelMA hydrogel concentration in bioartificial scaffolds was also optimized by static *in vitro* tests with HCFs to allow their long-term culture for at least 14 days. Finally, PCL/GelMA scaffolds cellularized with HCFs were cultured in dynamic conditions under cyclic mechanical stimulation to preliminarily assess the activation of HCFs into myofibroblasts.

### 3.1 Design and characterization of stretchable poly(*ε*-caprolactone) scaffolds

PCL scaffolds with cardiac tissue-like stretchability were designed to have a tailored mesh geometry with fixed filament size and a varying number of superimposed layers, to: (i) mimic the stiffness of human cardiac fibrotic tissues (Young’s modulus of 0.4–9 MPa, as measured by uniaxial tensile tests using a 500 N load cell);^28^ (ii) sustain *in vivo*-like maximum elastic deformation (≤22%);^5,21^ (iii) display fatigue resistance under uniaxial cyclic tensile tests (with 22% maximum strain).

**Figure 5:**
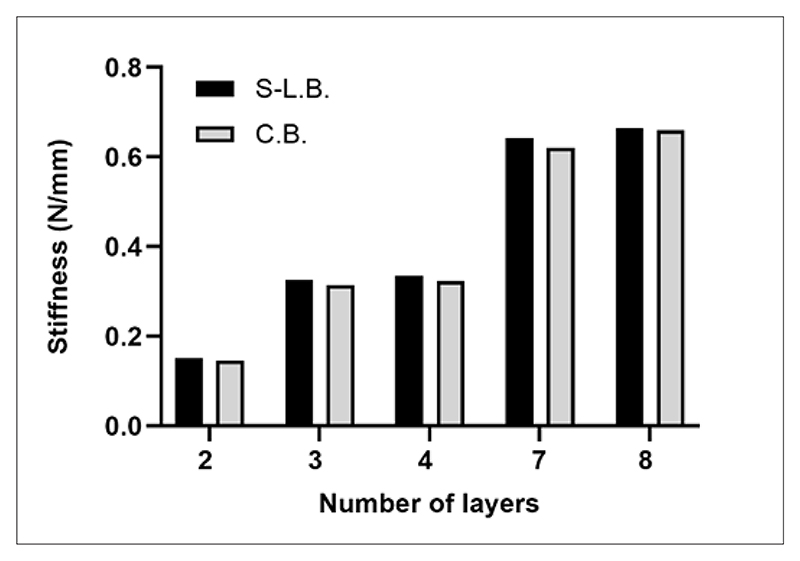
PCL: Poly(*ε*-caprolactone) (PCL) scaffold stiffness computed by structural analysis based on approximations of the single mesh element as a straight-line (S-L.B.) or a curved beam (C.B.). Abbreviations: C.B.: Curved beam; S-L.B.: Straight-line beam.

The scaffold mesh geometry was first defined (Figure 1), and PCL scaffolds with biomimetic stiffness were designed accordingly. Scaffolds with different numbers of layers (two, three, four, seven, and eight layers) were subjected to structural analysis under S-L.B. and C.B. hypotheses. Structure displacement computation was performed for stiffness analytic evaluation, considering both tensile force and bending moment (Figure S1). The analysis utilized Young’s modulus (*E*) and yield stress (*σ*_*YS*_) values of single PCL filaments fabricated by MEX that were measured by uniaxial tensile mechanical tests (*E* = 444 ± 46.32 *MPa* and *σ*_*YS*_ = 17.15 ± 2.36 *MPa*). Figures 5 and 9A displayed the stiffness of PCL scaffolds as a function of the number of scaffold layers, calculated according to the S-L.B. and C.B. approximations. Considering the PCL single filament section area (*b* × *h*) of 0.2 × 0.15 mm^2^, measured from SEM images (not shown), scaffold stiffness was found to increase as a function of the number of scaffold layers containing wavy filaments. Specifically, stiffness values increased twofold with the addition of any new layer containing wavy filaments, while layers containing straight filaments did not affect scaffold stiffness. This was a consequence of the dependence of the area moment on the scaffold cross-section. Indeed, the scaffold cross-section perpendicular to the *x*-direction (Figure 1) increased with the addition of any new layer containing wavy filaments. Hence, scaffolds with three, four, seven, and eight layers, that is, having the same number of layers with wavy filaments, displayed the same stiffness. As similar results were obtained using both S-L.B. and C.B. hypotheses (Figure 5), the S-L.B. approximation was then selected for further stiffness evaluations.

**Figure 6:**
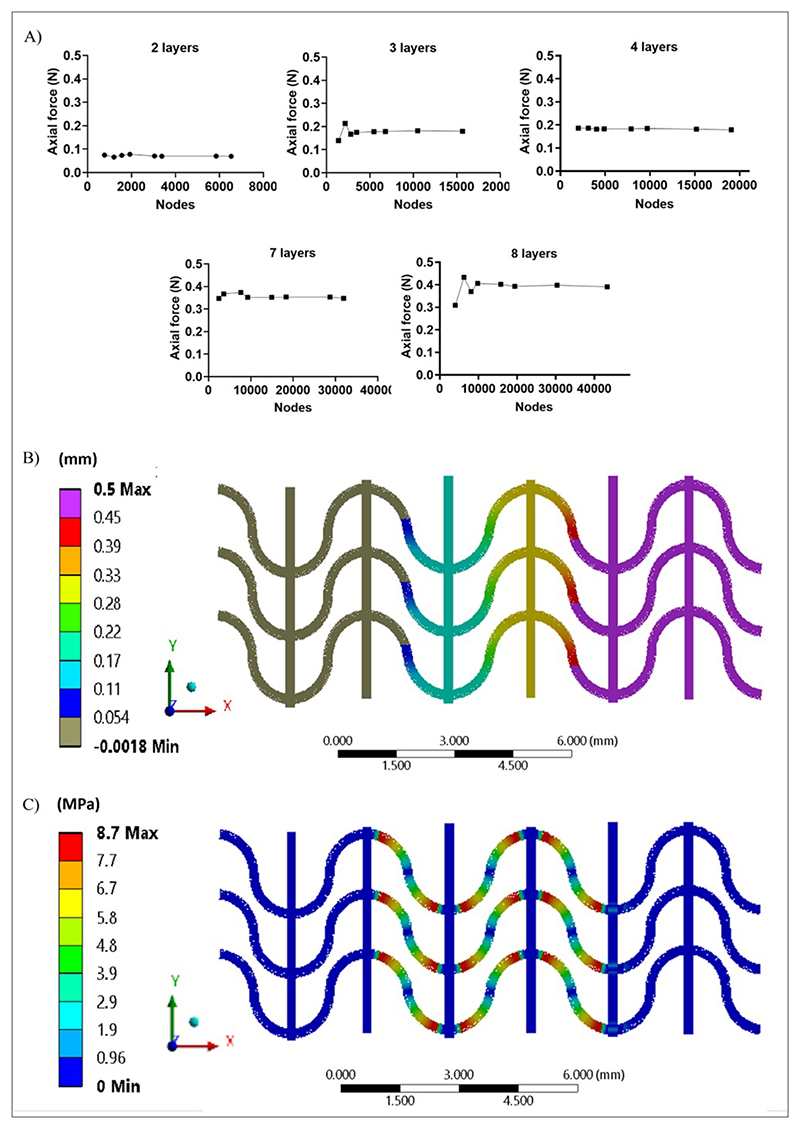
FEM analysis results (A) FEM mesh convergence study; (B) applied displacement values retrieved from numerical simulations; (C) Von Mises stress distribution for PCL scaffolds with two layers. Abbreviations: FEM: Finite element method; PCL: Poly(*ε*-caprolactone).

The mechanical behavior (e.g., stress distribution) of PCL scaffolds with up to eight stacked layers (Figure 1) was investigated by FEM analysis. Initially, a mesh convergence study was performed: Figure 6A reports the axial force as a function of the number of nodes for scaffolds with a different number of layers, indicating an asymptotic behavior. On this basis, a mesh was selected, ensuring acceptable accuracy (i.e., <2% variation with respect to the previously computed value) and preventing computational power waste. Simulations were run by imposing the selected displacement with a ramped profile, setting 100 displacement–force data points, and obtaining the configuration as displayed in Figure 6B.

Force values below yield stress conditions were obtained through a numerical algorithm for each of the imposed displacement points. Linear regression of the data was performed (Figure 6A), and the stiffness of PCL scaffolds with different thicknesses was computed as the angular coefficient of the curves displayed in Figure S2A. The evaluation of Von Mises stresses also enabled the validation of the elastic field hypothesis. Figure 6C illustrates an exemplary representation of Von Mises stress distributions for PCL scaffolds with two layers. Scaffolds with a different number of layers exhibited a similar distribution of Von Mises stresses. Qualitative considerations could be derived from the stress field of the 1D model: stress was distributed along the wavy filaments and the imposition of a bonded contact between straight and wavy filaments concentrated stress in proximity of such constraints regardless of the extinction zones present. The straight filaments were mostly unstressed and acted as load propagators in the direction of the deformation.

**Figure 7:**
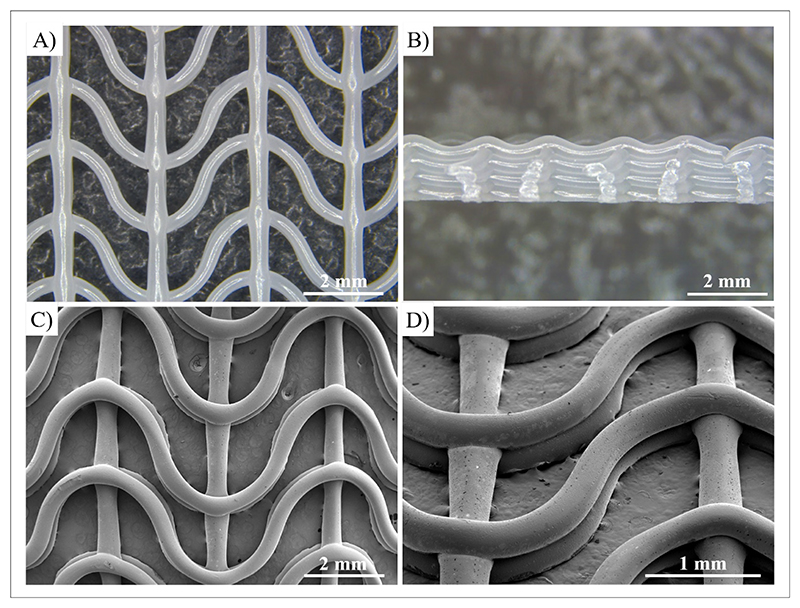
Optical images of the (A) top surface and (B) cross-section of an eight-layered PCL scaffold. SEM images of the (C) top surface and (D) tilted view of a three-layered PCL scaffold. Abbreviations: PCL: Poly(*ε*-caprolactone); SEM: Scanning electron microscopy.

PCL scaffolds were then fabricated by MEX, and their morphology was analyzed by optical microscopy and SEM. Optical microscopy (Figure 7A and B) and SEM images (Figure 7C and D) of PCL scaffolds with eight layers demonstrated that the scaffold geometry accurately reproduced the designed CAD model. The images revealed an inter-fiber distance of close wavy and straight filaments of 1.15 ± 0.12 and 1.63 ± 0.07 mm, respectively, compared to the corresponding theoretical values of 1.5 and 2 mm, respectively, in the CAD model. ImageJ analysis of the cross-section optical images (Figure 7B) reported that the cross-section of joint wavy filaments had an average width (*b*) of 0.22 ± 0.02 mm and an average height (*h*) of 0.43 ± 0.02 mm.

### 3.2 Gelatin methacryloyl hydrogels: Physicochemical characterization and *in vitro* cell tests

**Figure 8:**
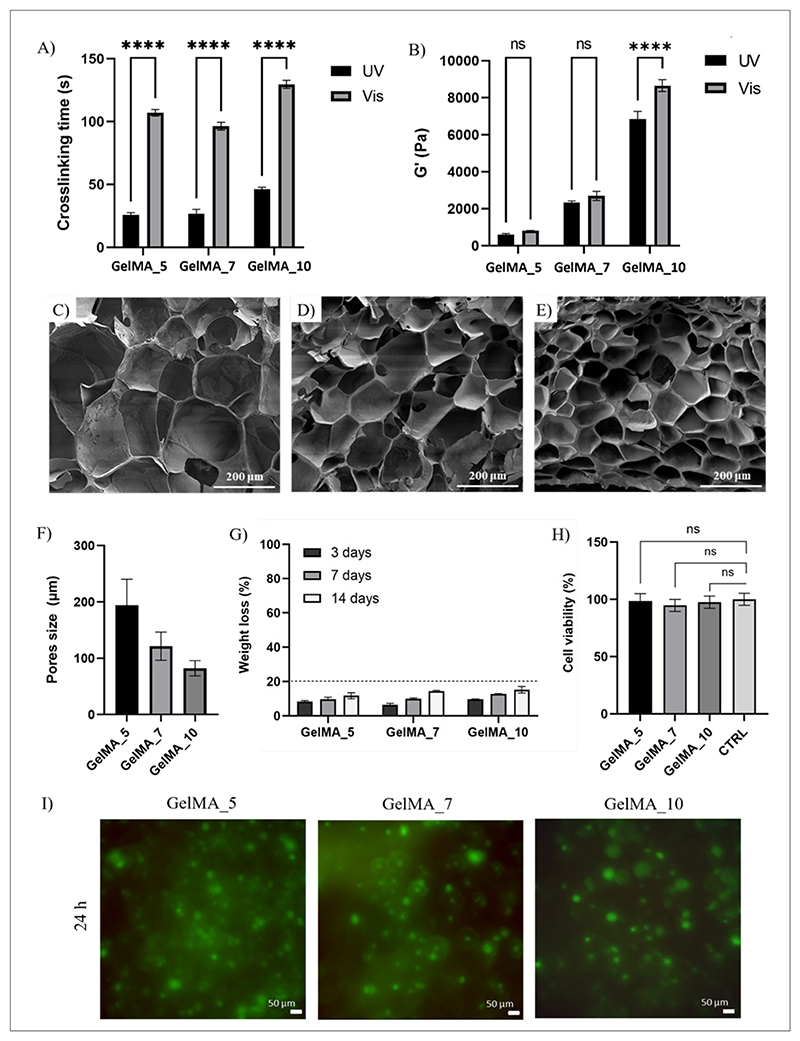
Gelatin methacryloyl (GelMA) hydrogel characterization (A) crosslinking time and (B) plateau onset values of storage modulus (G’) for GelMA_5, GelMA_7, and GelMA_10 hydrogels, exposed to ultraviolet (UV) and visible (Vis) light, measured from time-sweep curves (1% strain amplitude and 1 Hz frequency). ****p < 0.0001. (C–E) Scanning electron microscopy (SEM) images of freeze-dried samples: (C) GelMA_5, (D) GelMA_7, and (E) GelMA_10. (F) Pore size distribution of the freeze-dried samples and (G) weight loss percentage of GelMA_5, GelMA_7, and GelMA_10 hydrogels incubated in PBS at 37 °C. (H) Viability (CellTiter-Blue®) of cultured human cardiac fibroblasts (HCFs) in a culture medium containing eluates from GelMA_5, GelMA_7, and GelMA_10 hydrogels after 24 h incubation. (I) Live/Dead Cell Viability assay (green: live cells, red: dead cells) performed on HCF-cellularized GelMA hydrogels cultured for 24 h and cured accordingly (i.e., 30 s for GelMA_5 and 45 s for both GelMA_7 and GelMA_10 samples). Abbreviation: ns: Non-significant.

GelMA hydrogel crosslinking kinetics was first analyzed by rheological characterization of GelMA solutions in the presence of LAP photoinitiators using two different light sources (UV and Vis). The sol-gel transition of the GelMA_5, GelMA_7, and GelMA_10 samples (occurring when G’ exceeds G”) was measured at 37°C during UV or Vis light irradiation. Strain sweep tests performed on GelMA_5, GelMA_7, and GelMA_10 hydrogels reported that the 1% strain amplitude used in time sweep tests was within the linear viscoelastic region (curves not shown). Upon irradiation, *G*′ rapidly increased due to the photo-crosslinking process and reached a plateau at complete gelation.^28,29^ The crosslinking time (defined as the time *G*′ takes to reach the plateau value) for each GelMA hydrogel is reported in Figure 8A. At 37 °C and without irradiation, GelMA solutions retained a sol state as a function of time (data not shown). Under both UV and Vis irradiation, the crosslinking time increased as a function of GelMA solution concentration due to the presence of more reactive sites and increased viscosity.^35^ UV-photopolymerization was complete within approximately 20 s for GelMA_5, 25 s for GelMA_7, and 40 s for GelMA_10, while the crosslinking time for Vis-photopolymerized hydrogels was approximately 70 s for GelMA_5, 85 s for GelMA_7, and 100 s for GelMA_10. As the UV–vis spectrum of LAP photoinitiators has a main absorption peak at 365 nm, GelMA_5, GelMA_7, and GelMA_10 solutions exposed to UV light reported faster photo-crosslinking than under Vis light irradiation. To prepare the hydrogels, curing times slightly higher than measured crosslinking times were selected: (i) UV irradiation: 30 s for GelMA_5 and GelMA_7 and 45 s for GelMA_10; (ii) Vis light irradiation: 100 s for GelMA_5 and GelMA_7 and 120 s for GelMA_10. Rapid UV-photocrosslinking is advantageous for embedding cells into hydrogels as it minimizes cell exposure to UV irradiation.^36^ Biocompatibility was also demonstrated by Live/Dead analysis (Figure 8I) with no dead cells in GelMA hydrogels crosslinked under UV irradiation (30 s for GelMA_5 and GelMA_7 and 45 s for GelMA_10) and cultured for 24 h post-UV treatment.

*G*′ plateau onset values reported in Figure 8B increased with increasing hydrogel concentration^37^ from 1 to 8 kPa, and the values were higher for samples crosslinked under UV than Vis light irradiation. Interestingly, *G*′ values of GelMA hydrogels prepared from sterile and non-sterile solutions were also similar (Figure S3). Hence, the sterilization process, based on UV irradiation of solutions before photo-crosslinking, did not affect the rheological properties of GelMA hydrogels. The selected degree of methacrylate substitution (60%) and hydrogel concentrations (5–10% w/v) limited hydrogel crosslinking and entanglement, leading to *G*′ values in the range of stiffness of healthy cardiac tissue (1–40 kPa).^38^ However, the stiffness of the final bioartificial scaffolds was higher than that of hydrogels alone due to the reinforcing effect of PCL scaffold structures, depending on the number of scaffold layers.

UV-photocrosslinking was selected for the preparation of cell-embedded GelMA hydrogels, as it provided hydrogels with superior *G*′ upon brief exposure to UV irradiation, avoiding toxic effects on cells (Figure 8I).^36^ SEM images of fractured sections of freeze-dried UV-photocrosslinked GelMA_5, GelMA_7, and GelMA_10 hydrogels (Figure 8C–E) revealed increased pore sizes with decreasing GelMA concentration^38^ (Figure 8F). Figure 8G reports the weight loss of UV-photocrosslinked GelMA_5, GelMA_7, and GelMA_10 hydrogels incubated in sterile PBS at 37 °C for 14 days. Volumes of GelMA hydrogels and PBS were chosen at a 1:2 ratio (0.2 mL:0.4 mL). After 3 days, weight loss was less than 10% for all hydrogels, in agreement with previous reports.^39^ All samples had a weight loss under 20% after 14 days of incubation with no significant differences between the samples of different concentrations.

When embedding cells into GelMA hydrogels, free radicals could cause cytotoxicity, both directly or indirectly through the generation of reactive oxygen species (ROS). Furthermore, LAP has been reported to induce dose-dependent cytotoxic effects.^37,41^ To assess GelMA cytocompatibility, HCF viability was evaluated 24 h after culturing in hydrogel eluates (Figure 8H). Results indicated that the cell viability percentage was similar to control conditions (100%), that is, 98.9 ± 6.5%, 95.2 ± 5.3%, and 97.9 ± 5.4% for cells in contact with extracts from GelMA_5, GelMA_7, and GelMA_10 hydrogels, respectively. Hence, the hydrogels did not elicit cytotoxic effects.

### 3.3 Bioartificial poly(*ε*-caprolactone)-gelatin methacryloyl scaffolds: physicochemical characterization and *in vitro* cell tests

A polyDOPA coating was applied on the PCL scaffold surface, using a protocol previously reported by the authors,^24^ to enhance the interaction between PCL filaments and GelMA hydrogels. Thereafter, GelMA hydrogels and PCL scaffolds were combined into bioartificial PCL/GelMA scaffolds and provided with ECM-like microenvironment (HCFs-adhesive GelMA hydrogel). The scaffold was able to sustain *in vivo*-like cyclic mechanical deformations (stretchable PCL scaffolds) and exhibit cardiac fibrotic tissue-like Young’s modulus (400 kPa to 9 MPa).^22,20^

#### 3.3.1 Tensile testing

**Figure 9:**
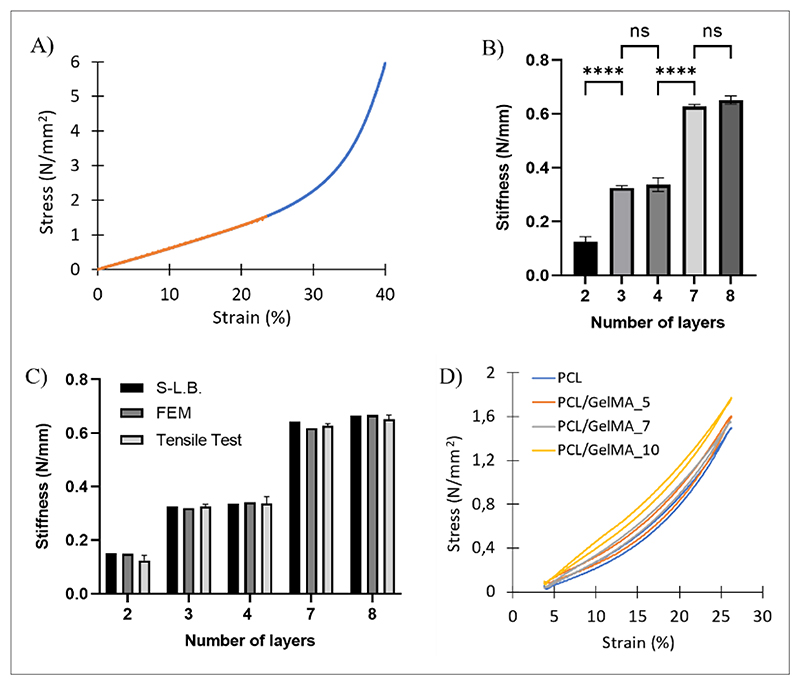
Stiffness and stress testing of the PCL scaffolds (A) PCL scaffold stress-strain curve; (B) Young’s modulus of PCL scaffolds with a different number of layers, calculated by tensile tests (****p < 0.0001); (C) stiffness of PCL scaffolds with a different number of layers, calculated by S-L.B. approximation, FEM analysis, and tensile tests; (D) cyclic stress-strain curves for PCL (blue), PCL/GelMA_5 (red), PCL/GelMA_7 (grey), and PCL/GelMA_10 (yellow) scaffolds (n = 3). Abbreviations: FEM: Finite element method; ns: Non-significant; PCL: Poly(*ε*-caprolactone); PCL/GelMA: Poly(*ε*-caprolactone)-gelatin methacryloyl; S-L.B: Straight-line beam.

PCL scaffolds, with 12 mm length, 4.5 mm width, and a thickness dependent on the number of layers, were subjected to tensile tests with and without GelMA_5, GelMA_7, and GelMA_10 hydrogel fillers to experimentally derive stiffness values (Figure 9 and Table 3). Stress–strain curves of PCL scaffolds displayed a J-shaped trend (Figure 9A).^6^ Stiffness values of PCL scaffolds with two, three, four, seven, and eight layers (Figure 9B) were comparable with those obtained by S-L.B. approximation and FEM analysis (Figure 9C). Stiffness increased with increasing the number of layers containing wavy filaments. The stiffness values (Figure 9B) and FEM outputs (Figure S2A) were similar for PCL scaffolds with three and four layers or with seven and eight layers. Hence, as aforementioned, the addition of a supplementary layer with straight filaments, perpendicularly oriented with respect to the load direction, did not vary scaffold stiffness. Scaffold mechanical response to the applied load was primarily due to the layers containing wavy filaments, while layers with straight filaments only behaved as strain distributors. Furthermore, PCL scaffolds with two to eight layers displayed a maximum elastic deformation equal to or superior to that of heart tissues at late-stage diastole (18–22%).^5,20^

The PCL scaffolds were functionalized with polyDOPA before the addition of GelMA hydrogels, obtaining PCL/GelMA scaffolds. Notably, polyDOPA coating did not affect the mechanical properties of PCL scaffolds, as previously reported for square grid-shaped scaffolds.^24^ PCL/GelMA scaffolds with four and eight layers were analyzed by tensile tests in wet conditions, and the stress–strain curves are reported in Figure S2B. The average stiffness and Young’s modulus values of the PCL/GelMA scaffolds, as derived from the tensile tests, are reported in Table 3.

Data collected in Table 3 indicated that stiffness and Young’s modulus of bioartificial PCL/GelMA scaffolds with a fixed number of layers slightly increased as a function of GelMA hydrogel concentration. At fixed GelMA hydrogel concentration, stiffness and Young’s modulus increased as a function of the number of layers. Young’s modulus of PCL/GelMA scaffolds was in the range of 6.8–10.5 MPa, which was in agreement with values reported in the literature for pathological cardiac tissues of men and women aged from 61 to 70.^19,20,38,43–45^ The literature reference values (i.e., between 400 kPa and 9 MPa) were derived from tensile tests performed on biopsies of human hearts extracted during autopsy.^5^ By increasing the concentration of GelMA hydrogel (and/or the number of scaffold layers), the mechanical properties of fibrotic human myocardium could be approached in terms of Young’s modulus (400 kPa to 9 MPa compared to 1–40 kPa for healthy cardiac tissue).^27,31,38^ For all bioartificial PCL/GelMA scaffolds, the maximum elastic deformation was equal to or slightly higher than the maximum elastic deformation of late-stage diastole (18–22%).^5,20,22^

Additionally, PCL/GelMA scaffolds were designed to exhibit dynamic stretchability for *in vitro* culture experiments under cardiac tissue-like cyclic mechanical stimulation (Figures 9D and S6 and Video S1). PCL scaffold geometry was mainly responsible for scaffold elastic stretchability. The maximum elastic deformation for PCL scaffolds was approximately 30% *versus* 3–4% for PCL dog-bone substrates and single filaments. Figure 9D reports one cycle of the cyclic mechanical test (100 cycles) displaying hysteretic behavior with structural damping. Hence, such preliminary tests suggested that PCL/GelMA scaffolds allowed cyclic mechanical deformation, while their stiffness could be adjusted by GelMA hydrogel concentration and the number of PCL scaffold layers.

#### 3.3.2 Biological evaluation

*In vitro* cell tests were performed on HCF-embedded GelMA hydrogels within bioartificial PCL/GelMA scaffolds with two layers, minimizing the use of cells. Cell viability, cytotoxicity, and immunofluorescence imaging were analyzed 7 and 14 days after cell culture.

**Figure 10:**
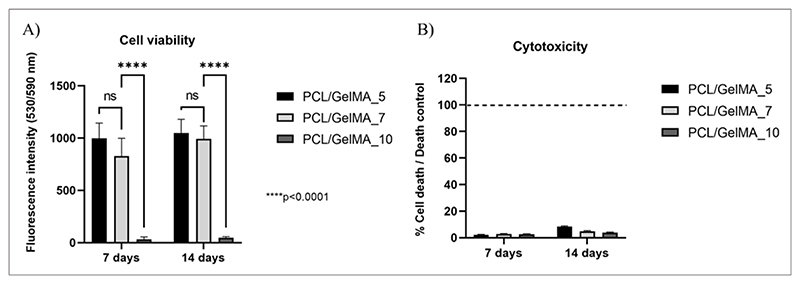
Biological evaluation of the PCL/GelMA scaffolds. (A) Cell viability percentage seven and 14 days after HCF culture in PCL/GelMA scaffolds with different GelMA concentrations (****p < 0.0001). (B) Cytotoxicity percentage seven and 14 days after HCF culture in PCL/GelMA scaffolds with different GelMA concentrations. The dashed line denotes the control cells; cultured HCFs displayed *p* < 0.0001 relative to the control cells. Abbreviations: GelMA: Gelatin methacryloyl; HCF: Human cardiac fibroblasts; PCL/GelMA: Poly(*ε*-caprolactone)-gelatin methacryloyl.

**Figure 11:**
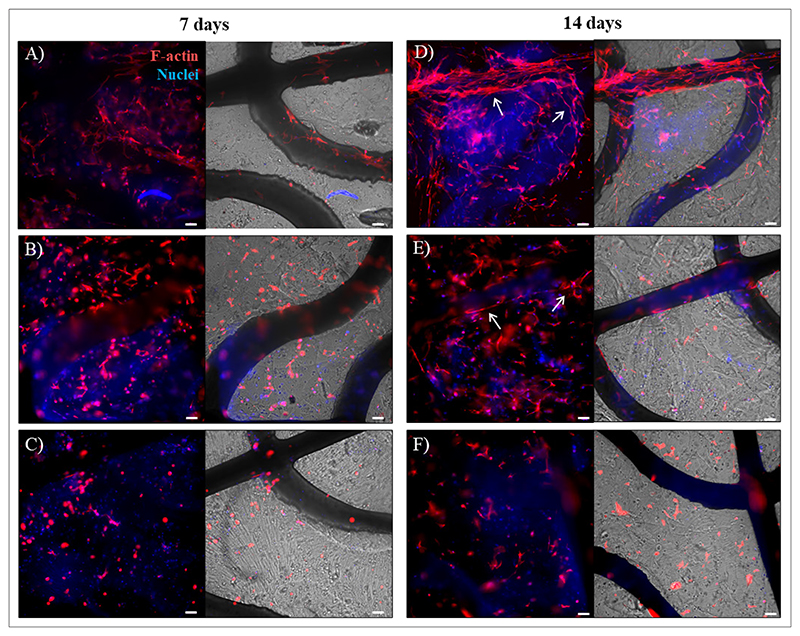
Immunofluorescence (red: F-actin; blue: nuclei) and bright-field images of cells and scaffolds after culture. HCFs on PCL/GelMA scaffolds with two layers seven days after HCF culture: (A) PCL/GelMA_5, (B) PCL/GelMA_7, and (C) PCL/GelMA_10. HCFs on PCL/GelMA scaffolds with two layers 14 days after HCF culture: (D) PCL/GelMA_5, (E) PCL/GelMA_7, and (F) PCL/GelMA_10. White arrows denote cell colonization in the PCL area. Scale bar: 100 µm. Abbreviations: HCF: Human cardiac fibroblasts; PCL/GelMA: Poly(*ε*-caprolactone)-gelatin methacryloyl.

No dead cells were observed in the Live/Dead images of GelMA_5, GelMA_7, and GelMA_10 hydrogels (Figure S4). However, cell viability (determined from the CellTiter-Blue® Cell Viability Assay) at days 7 and 14 was significantly higher for cells cultured in PCL/GelMA_5 and PCL/GelMA_7 compared to PCL/GelMA_10 scaffolds (Figure 10A). The high concentration of GelMA_10 hydrogel caused a reduction in its intrinsic porosity and permeability to nutrients and catabolites, affecting cell viability. This hypothesis was supported by SEM analysis of the freeze-dried hydrogel samples (Figure 8C–E) and previous findings.^34,42,46–47^ In contrast, the cytotoxicity assay did not reveal any cytotoxic effect on cells cultured on bioartificial scaffolds (Figure 10B). Cytotoxicity was evaluated by measuring lactate release in the medium from dead cells. Low cytotoxicity of cells cultured in PCL/GelMA_10 scaffolds suggested that HCFs populating the inner part of the hydrogel could be in a quiescent state. This hypothesis was supported by confocal microscopy analysis (Figures 11C and F and S5C and F) and by Live/Dead assay (Figure S4C and F), suggesting the presence of live cells with a rounded shape and less spread morphology within GelMA_10 hydrogels in PCL/GelMA_10 scaffolds even at 14 days after culture.

Cell F-actin and nuclei were stained to evaluate the 3D distribution of HCFs in the bioartificial scaffolds and their cytoskeleton morphology. Figures 11 and S5 report confocal microscopy images displaying a homogeneous distribution of cells inside the PCL/GelMA scaffolds and GelMA hydrogels, respectively, 7 and 14 days after culture. F-actin staining revealed a different cell cytoskeleton organization that depended on GelMA concentration, which is in agreement with previous literature,^4,37,48^ such that the hydrogels of lower concentrations and stiffness supported optimal cell spreading. Conversely, cells in the GelMA_10 hydrogel were rounded and not completely spread. Moreover, in PCL/GelMA_5 and PCL/GelMA_7 scaffolds, cells also colonized the PCL filaments (Figure 11D and E). PCL/GelMA_5 scaffolds were found to be the most suitable for HCF colonization of both PCL structure and GelMA hydrogel. Bright-field images were taken as a reference to localize the scaffold position in the hydrogel and to better inspect its effect on cell organization through confocal microscopy images. Overall, under static culture conditions, the presence of PCL scaffolds did not significantly influence cell orientation and distribution at the tested cell density.

**Figure 12:**
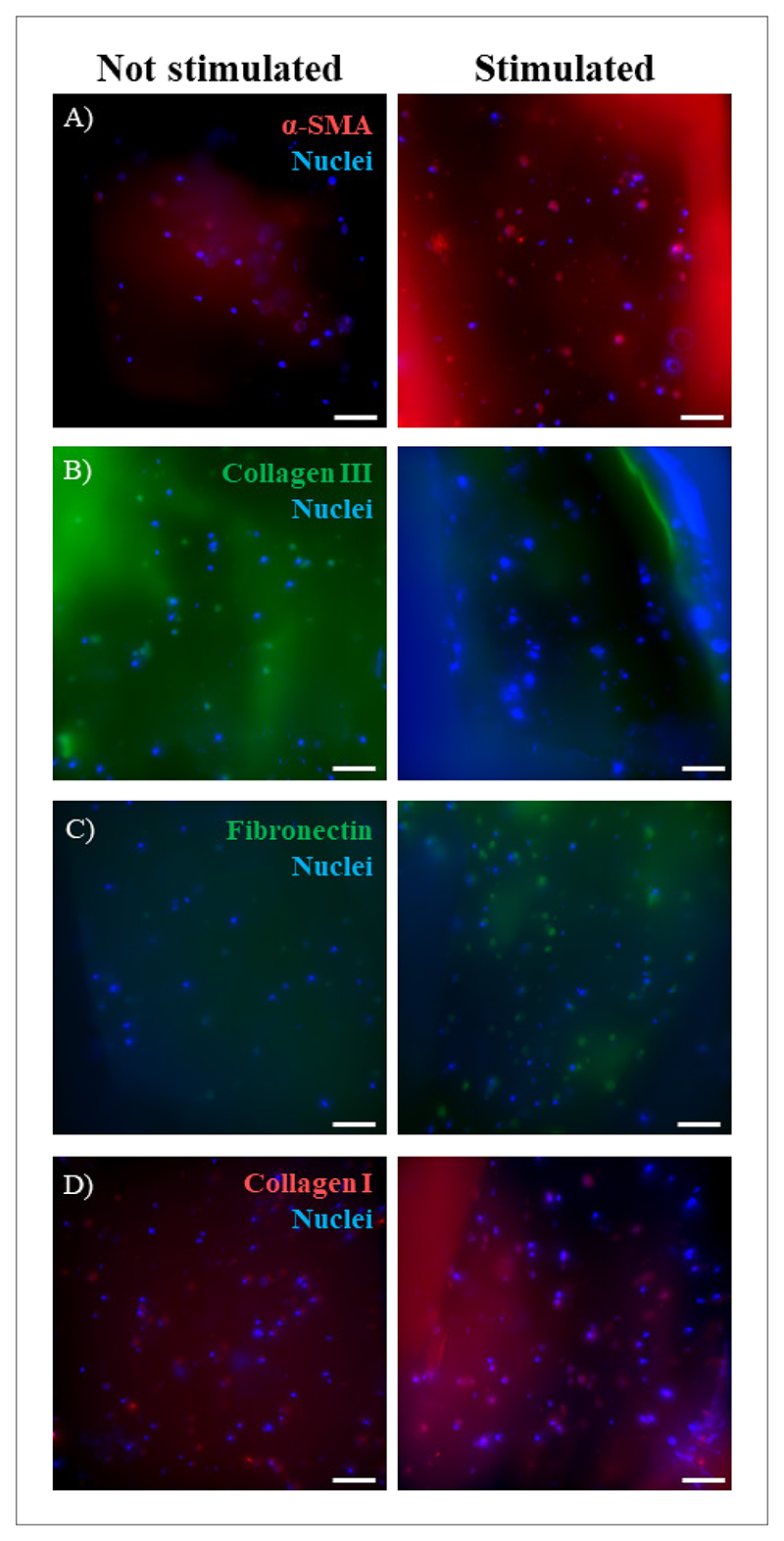
Immunostaining of HCFs on PCL/GelMA_5 scaffolds with and without mechanical cyclic stimulation (i.e., [left] not stimulated and [right] stimulated samples) for (A) *α*-SMA, (B) collagen III, (C) fibronectin, and (D) collagen I. Scale bar: 50 µm. Abbreviations: *α*-SMA: *α*-Smooth muscle actin; HCFs: Human cardiac fibroblasts; PCL/GelMA_5: Poly(*ε*-caprolactone)-gelatin methacryloyl.

Previous literature reported that chemical (e.g., TGF-*β*) or mechanical (e.g., stretching) pro-fibrotic stimuli^29,37^ can trigger *α*-SMA expression and modify the expression pattern of different ECM proteins, particularly the overexpression of fibronectin and collagen I and the downregulation of collagen III.^5,37,48^ Cyclic mechanical stimulation was applied by culturing HCF within PCL/GelMA_5 scaffolds in a MechanoCulture T6 bioreactor (under the experimental setup illustrated in Figure S6). Mechanical stimulation was applied 7 days after the initial culture in static conditions to allow cell attachment and spreading within GelMA_5 hydrogels (Figure 11A). Results revealed that mechanical stimulation triggered *α*-SMA overexpression in HCFs (Figure 12A), suggesting a phenotypic switch into myofibroblasts, of which was conversely not obtained in static culture conditions. Moreover, ECM protein staining indicated a decrease in the production of collagen III (Figure 12B), while fibronectin and collagen I were slightly more expressed after mechanical triggering (Figure 12C and D). Taken together, these results suggest that mechanical stimulation could favor pathological ECM deposition. However, these qualitative observations should be validated by quantifying protein production using immunoblotting and analyzing gene expression via real-time polymerase chain reaction (qPCR).^5,29^ Furthermore, longer stimulation periods could enhance tissue response to the mechanical stimulus, warranting future investigations to elucidate the role of mechanical stress in the onset of fibrosis.

Overall, the results suggested that bioartificial PCL/GelMA scaffolds cultured with HCFs can potentially recreate an *in vitro* cardiac fibrotic tissue model with *in vivo*-like cyclic stretching. Although miniaturized platforms (organ-on-chips) have been developed (e.g., to reproduce heartbeats),^29^ the advantage of a tissue-engineered cardiac scar model is represented by its potential exploitation to test not only drugs and nanotherapeutics, but also medical devices, cell therapies, and tissue engineering solutions.

## 4 Conclusion

In this work, 3D bioartificial stretchable scaffolds were designed, combining GelMA hydrogels and PCL scaffolds fabricated by MEX. Stretchability was imparted by PCL scaffold architecture, consisting of alternating layers of parallel wavy and straight filaments with 90° inclination with respect to the wavy filaments. Stiffness values of PCL scaffolds, obtained by structural and FEM analyses, were in agreement with experimental data derived by tensile tests: stiffness increased from 0.15 to 0.66 N/mm as a function of the number of layers (from two to eight), and only additional layers containing wavy filaments contributed to stiffness increase.

Photocrosslinked GelMA hydrogels were prepared at three different concentrations via visible or UV light crosslinking to determine optimal biomimetic support for embedding cells into bioartificial scaffolds. UV photocrosslinking and exposure time were selected based on photo-rheological characterization and Live/Dead assay. GelMA_5, GelMA_7, and GelMA_10 hydrogels exhibited biomimetic stiffness (1–8 kPa) comparable to healthy cardiac tissues. Moreover, sterilized hydrogels were stable for up to 14 days of incubation in sterile PBS. Their biocompatibility was assessed by *in vitro* cell tests on hydrogel eluates.

Before the PCL and GelMA combination, polyDOPA coating was applied on the PCL scaffolds to improve GelMA adhesion onto the PCL scaffold surface. Bioartificial PCL/GelMA scaffolds reported stiffness and Young’s modulus values of 0.51–1.18 N/mm and 6.8–10.5 MPa, respectively, and these parameters were dependent on the number of layers and GelMA hydrogel concentration. Nonetheless, Young’s modulus values were in agreement with literature data on pathological cardiac tissue for men and women between the ages of 61 and 70.^27^ Furthermore, bioartificial PCL/GelMA scaffolds displayed biomimetic elastic deformation of up to 22% strain.^25^

*In vitro* cell tests were performed by embedding HCFs into GelMA hydrogels, filling the pores of PCL/GelMA scaffolds with two layers. The cells were well-distributed throughout the hydrogel. Likewise, the cytoskeletal conformation was strongly influenced by GelMA hydrogel concentration, inferring better cell spreading within the PCL/GelMA_5 scaffold. Although all hydrogels were not cytotoxic, only GelMA_5 and GelMA_7 hydrogels supported the viability of embedded cells, probably due to their superior permeability to nutrients and metabolites. Moreover, cell-loaded PCL/GelMA_5 scaffolds mechanically stimulated with MechanoCulture T6 bioreactor reported higher expressions of proteins involved in fibrosis, compared to scaffolds under static conditions. In particular, stimulated cells had increased expressions of the *α*-SMA and fibronectin and decreased expression of collagen III.^48^ These results supported the hypothesis that the application of cyclic mechanical stimulations to cellularized bioartificial scaffolds may trigger HCF activation into myofibroblasts. Deeper investigations through protein and gene expression quantifications (e.g., qPCR) are warranted to validate the effect of mechanical stimulation on HCF phenotype and culture time. This system would allow us to better understand and manage fibrosis onset by tuning stretch timing and rate.

In summation, the mechanical and biological properties of bioartificial PCL/GelMA scaffolds make them a promising platform with *in vivo*-like mechanical properties, tailored by PCL scaffold architecture and GelMA hydrogel concentration, for the development of dynamic *in vitro* models of human cardiac fibrotic tissues. Further validation will allow their use for the *in vitro* preclinical testing of medical devices and advanced therapies.

## Supplementary Material

IJB2247 - Suppl. File - Final

IJB2247_video S1.mp4

## Figures and Tables

**Table 1:** Equations for stiffness computation for a single beam element, using the elastic line equation under S.-L.B. and C.B. approximations

Approximation type	Equation for stiffness calculation
S-L.B.	$K_{ij}^{SLB}=3EAI/\left(L\cdot \left(3I\;\text{sin}{\left(\alpha \right)}^{2}+AL^{2}\;\text{cos}{\left(\alpha \right)}^{2}\right)\right)$
C.B.	$K_{ij}^{CB}=12EAI/\left(R\cdot \left(3I\pi +AR^{2}\cdot (3\pi -8)\right)\right)$

Abbreviations: A: Cross-section area; alfa: Angle between the beam and vertical direction; **C.B.: Curved beam;** E: PCL Young’s Modulus;I: Moment of inertia associated with the cross section; L: Straight beam length;R: Curvature radius of curved beam; **S-L.B.: Straight-line beam**.

**Table 2:** Stiffness equations applied for calculating the total stiffness of stretchable PCL mesh

Stiffness for different samples	Equation for stiffness calculation
Series springs	$1/K_{i}=\underset{j}{\sum }1/K_{ij}$
Parallel springs	$K_{TOT\;}=\underset{i}{\sum }K_{i}$
Total 2D mesh	*K*_*mesh*_ = *K*_*TOT*_ = *K* · (*i / j*)

Note: K*ij* stiffness for a single element is constant and independent of *i*-*j* indexes. Abbreviations: K: stiffness; PCL: poly(*ε*-caprolactone); TOT: total.

**Table 3:** Stiffness and Young’s modulus values of PCL/GelMA scaffolds derived from uniaxial tensile tests in wet condition

Number of layers	Bioartificial scaffold	Tensile test	
		Stiffness (N/mm)	Young’s modulus (MPa)
4	PCL/GelMA_5	0.51 ± 0.01	6.76 ± 0.18
	PCL/GelMA_7	0.56 ± 0.01	7.66 ± 0.18
	PCL/GelMA_10	0.65 ± 0.10	9.47 ± 1.39
8	PCL/GelMA_5	0.85 ± 0.07	8.21 ± 0.68
	PCL/GelMA_7	0.96 ± 0.03	8.88 ± 0.26
	PCL/GelMA_10	1.18 ± 0.01	10.46 ± 0.03

Abbreviation: PCL/GelMA: poly(*ε*-caprolactone)-gelatin methacryloyl.

## Data Availability

Data are available from the corresponding author upon reasonable request.
