# Supplementary material for: 3D bioartificial stretchable scaffolds mimicking the mechanical hallmarks of human cardiac fibrotic tissue
Source: Int J Bioprint. Author manuscript; Available in PMC 2024 Sep 19. (PMC7616559; doi:10.36922/ijb.2247)
Supplement: IJB2247 - Suppl. File - Final [file EMS198444-supplement-IJB2247___Suppl__File___Final.pdf]

ACCSCIENCE PUBLISHING

International Journal of Bioprinting

RESEARCH ARTICLE

3D bioartificial stretchable scaffolds mimicking the mechanical hallmarks of human cardiac fibrotic tissue

Supplementary file

(A) Supporting information

Models for analytical stiffness computation

To evaluate the mechanical properties of stretchable poly(ε-caprolactone) (PCL) scaffolds, stiffness was initially estimated by an analytical approach, varying the number of scaffold layers and, consequently, scaffold thickness.

Structural analysis of scaffold stiffness was performed on the repeating half-semicircle element of the wavy pattern, approximating it with a straight-line beam (S-L.B.) (dashed line) or a curved beam (C.B.) (red portion or the half-semicircle) (Figure S1). The curved element was approximated with an S-L.B. inclined at α angle and having L length. A fixed support constraint was placed at one end of the beam (A), while the other free end of the beam (B) was subjected to F load along the x-direction.

The simple scheme of a cantilever beam being subjected to tensile and bending load was considered. Elastic line equations were employed to obtain displacements along the x' ($u_{x'}$) and y' ($v_{y'}$) axes. The expressions of displacement components $u_{x'}$ (**Equation SI**) and $v_{y'}$ (**Equation SII**) were obtained through the application of a rotation matrix, comprising both the inclination of the beam axis and the variation of loading direction.

$$u_{x'} = \frac{F \cdot L}{E \cdot A} \tag{SI}$$

$$v_{y'} = \frac{F \cdot L^3}{3E \cdot I} \tag{SII}$$

The displacement components $u_{x'}$ and $v_{y'}$ were then combined to obtain the expression of the displacement δ^{SLB}:

$$\delta^{SLB} = \frac{F \cdot L}{E} \cdot \left(\frac{1}{A} \cdot \sin^2(\alpha) + \frac{L^3}{3I} \cdot \cos^2(\alpha) \right) \tag{SIII}$$

where L is the length of the S-L.B., E is the elastic modulus of the material, A (b × h) is the cross-sectional area, I is the area moment associated with the cross-section, and α is the inclination of the beam axis with respect to the vertical axis.

The curved element was considered as C.B. (Figure S1). To identify positions along the beam axis, a polar reference frame along x'–y' was introduced, considering radius R and the angular coordinate θ. This additional reference frame was used to express internal loads (i.e., tensile contribution [N], shear contribution [T], and bending moment [M]). The overall δ^{CB} displacement for C.B. along the x-direction was calculated by combining the displacements due to tensile and bending loads as follows:

$$\delta^{CB} = \frac{F \cdot R}{E \cdot A} \cdot \frac{\pi}{4} + \frac{F \cdot R^3}{E \cdot I} \cdot \left(\pi - \frac{8}{3} \right) \tag{SIV}$$

where R is the radius of curvature of the beam element.

Both expressions for displacement (**Equations SIII** and **SIV**), as a function of F, were used to calculate stiffness (K). As displayed in **Figure 5**, both approximations provided comparable stiffness values. For this reason, the S-L.B. approximation was selected for further stiffness evaluations.

Mechanical behavior within the elastic range of deformation

For PCL scaffolds with different number of layers, force–displacement curves were obtained by tensile tests and finite element method (FEM) analysis. Figure S2 displays the force–displacement curves in the linear region until 1-mm displacement, that is, within the strain reference values of 15–22%. Figure S2A compares the force–displacement curves for scaffolds with a different number of layers, and the curves exhibit a similar behavior, demonstrating the accuracy of FEM simulation. Figure S2B displays the linear region of force–displacement curves obtained from tensile tests conducted on

bioartificial poly(ε-caprolactone)-gelatin methacryloyl (PCL/GelMA) scaffolds. For bioartificial scaffolds with four layers, stiffness was approximately independent of GelMA hydrogel concentration. For bioartificial scaffolds with eight layers, slight changes in stiffness were recorded as a function of GelMA hydrogel concentration. On the contrary, changes in the number of layers greatly affected bioartificial scaffold stiffness.

Rheological characterization of sterile gelatin methacryloyl hydrogels

Rheological characterization was performed on GelMA solutions prepared from sterile (sterilization protocol described in Section **2.11. Long-term cell viability and cytotoxicity tests on bioartificial stretchable scaffolds**) and not sterile solutions to evaluate the effect of ultraviolet (UV)-sterilization on hydrogel crosslinking kinetics. Results revealed that both curing time and G' were similar, suggesting that sterilization did not affect the rheological properties of GelMA hydrogels (Figure S3).

Biological validation of gelatin methacryloyl hydrogels

Live/Dead assay was performed 7 and 14 days after culture to confirm cell integration and viability in 3D GelMA hydrogels. Figure S4 revealed no dead cells in all types of tested GelMA hydrogels (GelMA_5, GelMA_7, and GelMA_10).

Human cardiac fibroblast (HCF) distribution in the bioartificial scaffolds and their cytoskeletal morphology were evaluated through F-actin and nuclei fluorescent staining. Furthermore, α-smooth muscle actin (α-SMA) was stained to evaluate the fibrotic phenotype change in HCFs. Figure S5 reports a homogeneous distribution of HCFs within GelMA hydrogels 7 and 14 days after culture. However, α-SMA was not expressed by HCFs within GelMA hydrogels 14 days after culture, suggesting that the phenotypic switch of HCFs to myofibroblasts did not occur in static conditions.

Mechanical stretching in bioreactor

The MechanoCulture T6 bioreactor (CellScale, Canada) experimental setup is illustrated in Figure S6. PCL meshes with 4.5 × 1.5 unit cells in the x–y plane (corresponding to 18 × 4.5 mm^2), a gauge length of 1.5 unit cells along the x-axis (corresponding to 6 mm), and four layers of scaffolds were kept in motion (Video S1), and the scaffolds were subsequently subjected to cyclic mechanical deformations up to 10% maximum deformation at 1 Hz frequency for 5 days prior to testing their mechanical behavior.

(B) Supplementary tables and figures

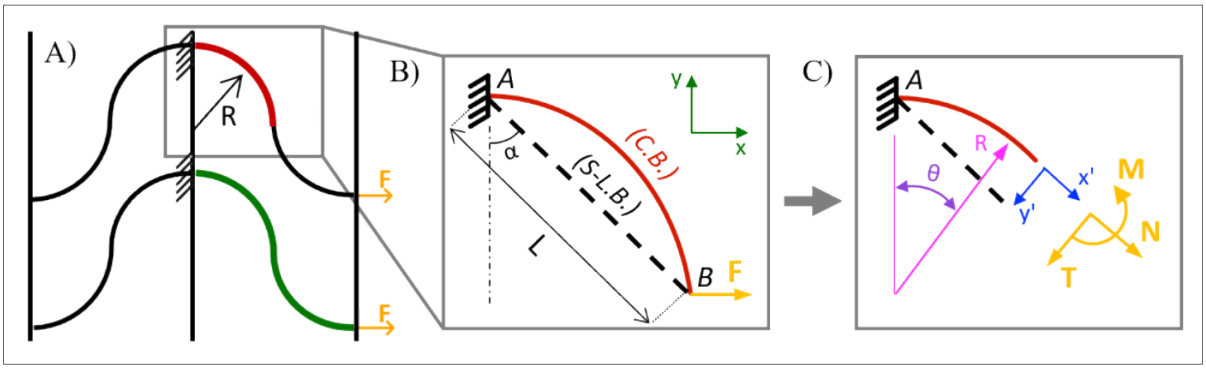

Figure S1. Models for analytic stiffness computation. (A) Straight-line beam (S-L.B.) and curved beam (C.B.) approximations of the repeating half-semicircle element of the wavy pattern. For both approximations, internal action schemes are reported at the (B) global cartesian reference (x–y) and (C) local Cartesian reference frame (x'–y') and internal actions (N, M, T).

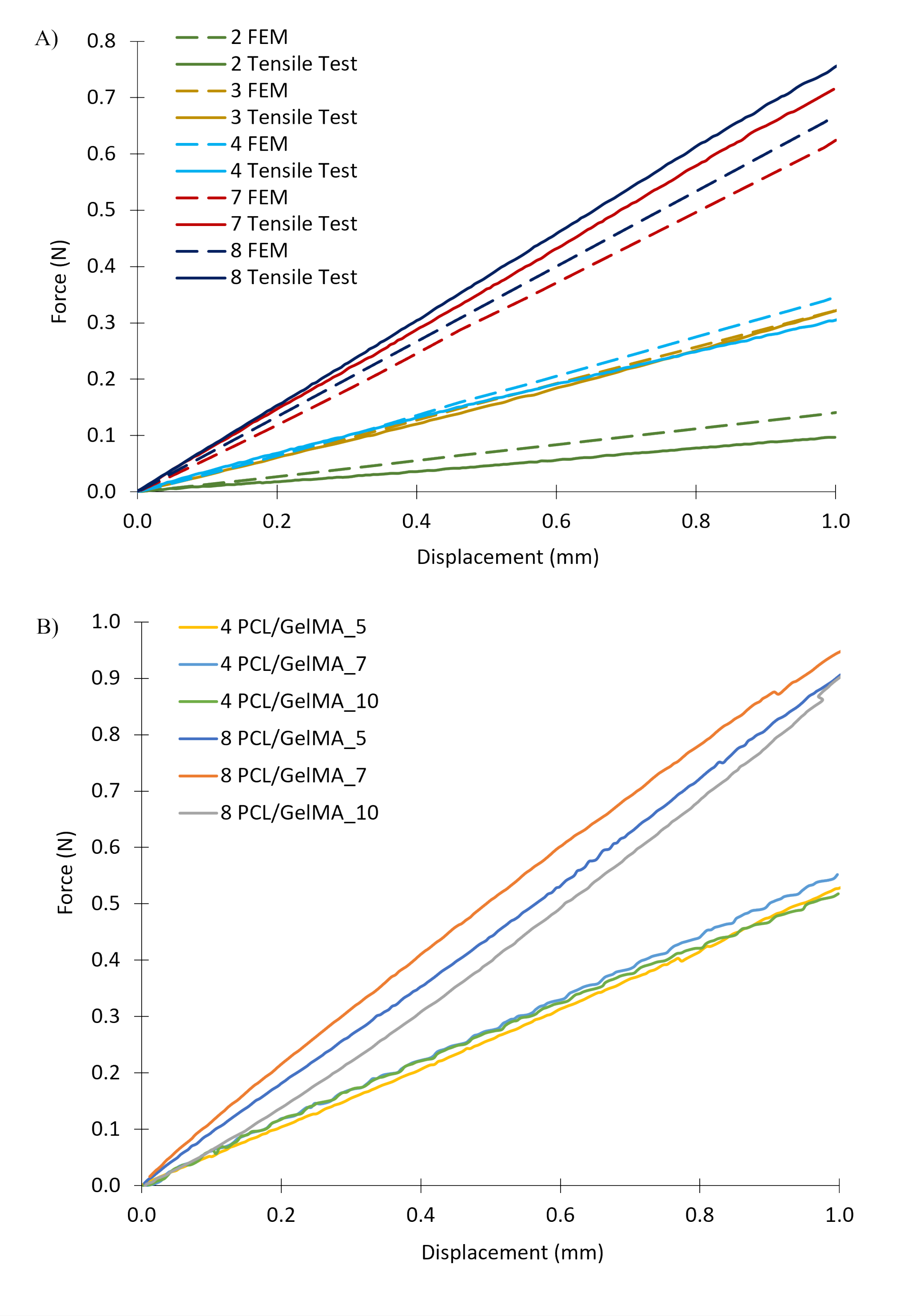

Figure S2. Force–displacement curves within the linear region, corresponding to the elastic range of deformations: (A) for bioartificial scaffolds with different number of layers (two, three, four, seven, and eight layers), obtained by FEM simulation and tensile tests; (B) for PCL/GelMA scaffolds (four and eight layers) containing GelMA_5, GelMA_7, and GelMA_10 hydrogels, obtained by tensile tests. Abbreviations: FEM, finite element method; PCL/GelMA, poly(ε-caprolactone)-gelatin methacryloyl.

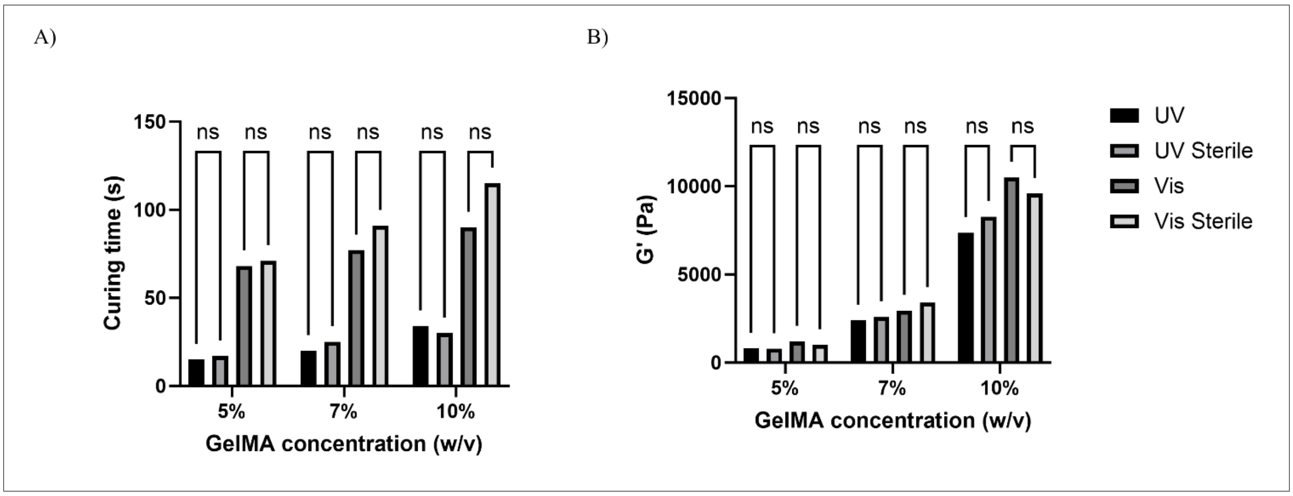

Figure S3. Rheological characterizations of GelMA hydrogels prepared from non-sterile and sterile solutions: (A) crosslinking time and (B) storage modulus (G′) of GelMA_5, GelMA_7, and GelMA_10, exposed to UV and Vis light from time sweep tests using 1% strain amplitude and 1 Hz frequency. Abbreviations: GelMA, gelatin methacryloyl; ns, nonsignificant; UV, ultraviolet; vis, visible.

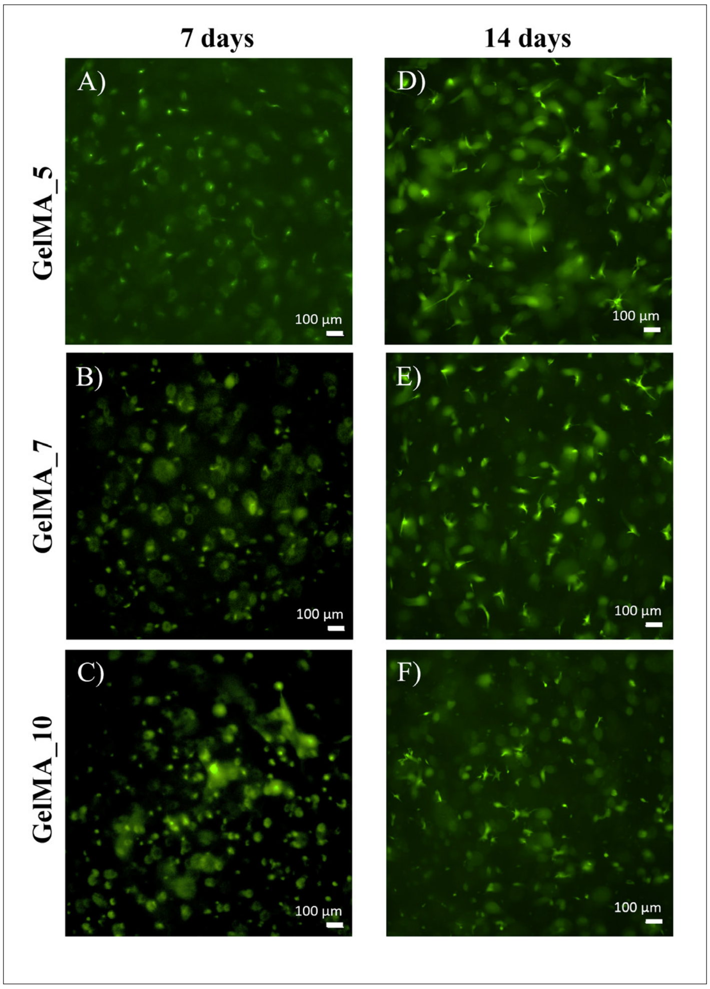

Figure S4. Live/dead assay (green: live cells; red: dead cells) of HCFs cultured within GelMA hydrogels after 7 and 14 days of culture. On the left column: (A) GelMA_5, (B) GelMA_7, and (C) GelMA_10 after 7 days of culture. On the right column: (D) GelMA_5, (E) GelMA_7, and (F) GelMA_10 after 14 days of culture. Abbreviations: GelMA, gelatin methacryloyl; HCF, human cardiac fibroblast.

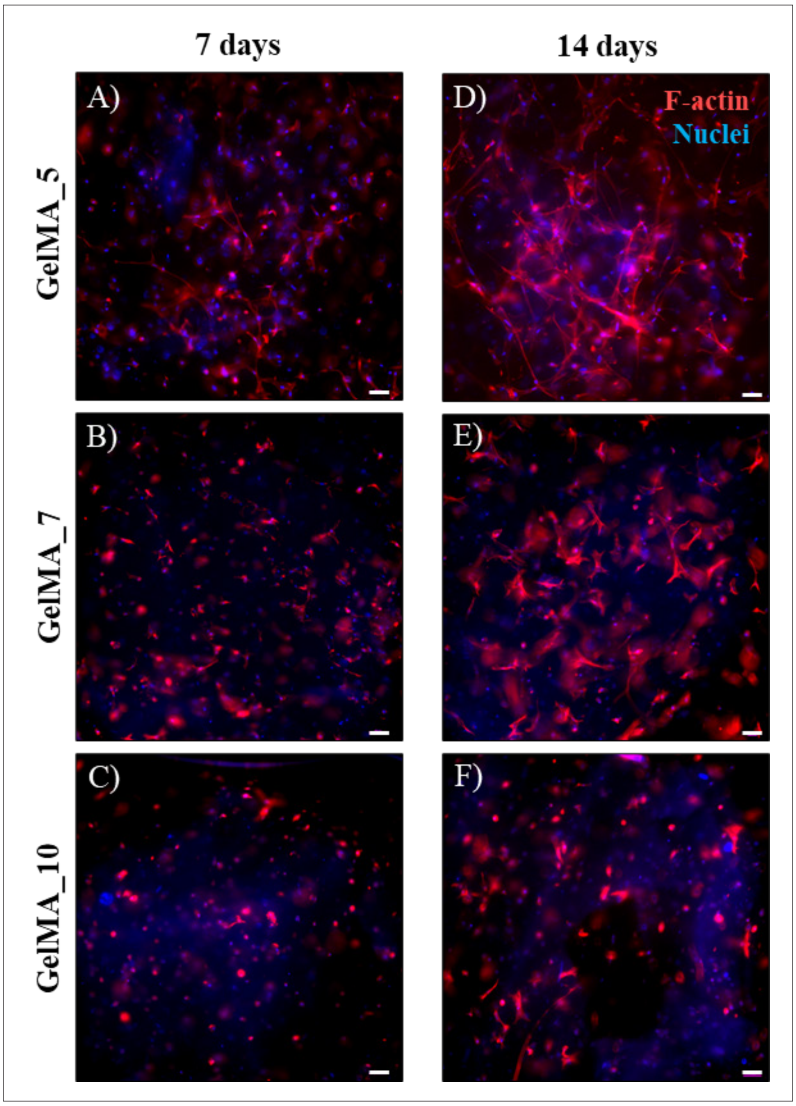

Figure S5. Immunofluorescence images (red: F-actin; blue: nuclei) of HCFs cultured within GelMA hydrogels after 7 and 14 days of culture. On the left column: (A) GelMA_5, (B) GelMA_7, and (C) GelMA_10 after 7 days of culture. On the right column: (D) GelMA_5, (E) GelMA_7, and (F) GelMA_10 after 14 days of culture. Scale bar: 100 μm. Abbreviations: GelMA, gelatin methacryloyl; HCF, human cardiac fibroblast.

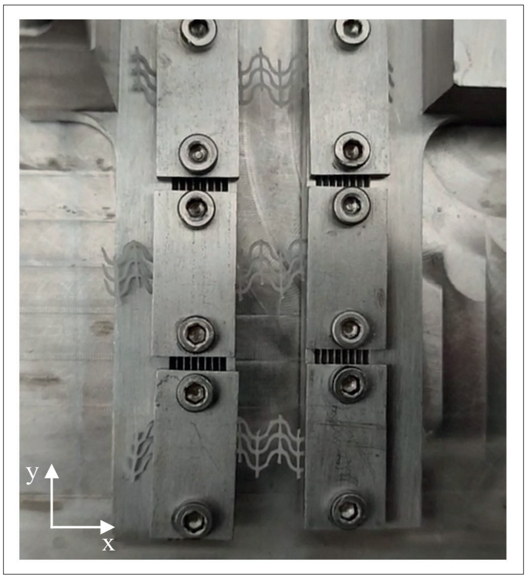

Figure S6. The setting of PCL mesh in the MechanoCulture T6 bioreactor (CellScale, Canada) for preliminary cyclic fatigue mechanical testing. Abbreviation: PCL, poly(ε-caprolactone).

(C) Supplementary video

Video S1. Functioning of MechanoCulture T6 bioreactor (CellScale, Canada) for preliminary cyclic fatigue mechanical testing of poly(ε-caprolactone) (PCL) meshes, stretched at 1 Hz with 10% deformation.
